# RETRACTION: A Meta‐Analysis Showing the Effect of Surgical Site Wound Infections and Associated Risk Factors in Neonatal Surgeries

**DOI:** 10.1111/iwj.70416

**Published:** 2025-03-26

## Abstract

**RETRACTION:** AbdelgawadM. A. , ParambiD. G. T. , GhoneimM. M. , AlotaibiN. H. , AlzareaA. I. , AlanaziA. S. , HassanA. , TonyS. M. , and AbdelrahimM. E. A. , “A Meta‐Analysis Showing the Effect of Surgical Site Wound Infections and Associated Risk Factors in Neonatal Surgeries,” International Wound Journal 19, no. 8 (2022): 2092–2100, 10.1111/iwj.13814.35445789 PMC9705165

The above article, published online on 21 April 2022, in Wiley Online Library (http://onlinelibrary.wiley.com/), has been retracted by agreement between the journal Editor in Chief, Professor Keith Harding; and John Wiley & Sons Ltd. Following an investigation by the publisher, all parties have concluded that this article was accepted solely on the basis of a compromised peer review process. The editors have therefore decided to retract the article. The authors did not indicate their agreement with the retraction.

**RETRACTION:** M. A. Abdelgawad , D. G. T. Parambi , M. M. Ghoneim , N. H. Alotaibi , A. I. Alzarea , A. S. Alanazi , A. Hassan , S. M. Tony , and M. E. A. Abdelrahim , “A Meta‐Analysis Showing the Effect of Surgical Site Wound Infections and Associated Risk Factors in Neonatal Surgeries,” International Wound Journal 19, no. 8 (2022): 2092–2100, 10.1111/iwj.13814.35445789 PMC9705165

The above article, published online on 21 April 2022, in Wiley Online Library (http://onlinelibrary.wiley.com/), has been retracted by agreement between the journal Editor in Chief, Professor Keith Harding; and John Wiley & Sons Ltd. Following an investigation by the publisher, all parties have concluded that this article was accepted solely on the basis of a compromised peer review process. The editors have therefore decided to retract the article. The authors did not indicate their agreement with the retraction.

